# Methylotroph bacteria and cellular metabolite carotenoid alleviate ultraviolet radiation-driven abiotic stress in plants

**DOI:** 10.3389/fmicb.2022.899268

**Published:** 2023-01-06

**Authors:** Santosh Ranjan Mohanty, Himanshu Mahawar, Apekcha Bajpai, Garima Dubey, Rakesh Parmar, Nagvanti Atoliya, Mayanglambam Homeshwari Devi, Amar Bahadur Singh, Devendra Jain, Ashok Patra, Bharati Kollah

**Affiliations:** ^1^ICAR-Indian Institute of Soil Science, Bhopal, Madhya Pradesh, India; ^2^ICAR -s Directorate of Weed Research, Jabalpur, Madhya Pradesh, India; ^3^Department of Molecular Biology and Biotechnology, Maharana Pratap University of Agriculture and Technology, Udaipur, Rajasthan, India

**Keywords:** methylotroph, carotenoid, UV irradiation, plant-microbe interaction, beta carotene

## Abstract

Increasing UV radiation in the atmosphere due to the depletion of ozone layer is emerging abiotic stress for agriculture. Although plants have evolved to adapt to UV radiation through different mechanisms, but the role of phyllosphere microorganisms in counteracting UV radiation is not well studied. The current experiment was undertaken to evaluate the role of phyllosphere *Methylobacteria* and its metabolite in the alleviation of abiotic stress rendered by ultraviolet (UV) radiation. A potential pink pigmenting methylotroph bacterium was isolated from the phylloplane of the rice plant (o*ryzae sativa*). The 16S *rRNA* gene sequence of the bacterium was homologous to the *Methylobacter sp*. The isolate referred to as *Methylobacter sp* N39, produced beta-carotene at a rate (μg ml^–1^ d^–1^) of 0.45–3.09. Biosynthesis of beta-carotene was stimulated by brief exposure to UV for 10 min per 2 days. Carotenoid biosynthesis was predicted as *y* = 3.09 × incubation period + 22.151 (*r*^2^ = 0.90). The carotenoid extract of N39 protected *E. coli* from UV radiation by declining its death rate from 14.67% min^–1^ to 4.30% min^–1^ under UV radiation. Application of N39 cells and carotenoid extract also protected *rhizobium* (*Bradyrhizobium japonicum*) cells from UV radiation. Scanning electron microscopy indicated that the carotenoid extracts protected *E. coli* cells from UV radiation. Foliar application of either N39 cells or carotenoid extract enhanced the plant’s (Pigeon pea) resistance to UV irradiation. This study highlight that *Methylobacter sp* N39 and its carotenoid extract can be explored to manage UV radiation stress in agriculture.

## Introduction

Solar ultraviolet (UV) radiation on the earth’s surface is increasing due to stratospheric ozone depletion, caused by an accelerated increase in greenhouse gas emission. Solar UV radiation is mainly three types: UV-A radiation (315–400 nm) leads to indirect damage to cellular DNA, proteins, and lipids by generating intracellular reactive oxygen species (Sage et al., 2012; Lefatshe et al., 2020). UV-B (280–315 nm) radiation causes direct DNA damage by inducing the generation of DNA photoproducts, such as pyrimidine dimers (Acuña-Rodríguez et al., 2021). The most energetic rays are the UV-C (100–280 nm) wavelengths, which has the highest potential to cause mutation and deleterious effect on living beings. Although the ozone layer efficiently filters out these UV-C rays, due to depletion of ozone layer UV-C radiation is increasing on the earth’s surface over the years. Therefore, both UV-B and UV-C on earth critically affecting plants and animals. Recently, it is observed that higher UV radiation is imposing a serious threat to agriculture (Barnes et al., 2015). UV radiation affects bacterial growth and metabolism for which the radiation is used for controlling bacteria. The UV-C exhibits the most effective germicidal effect on bacteria (Tran et al., 2022). In a study, the responses of two gram-negative bacteria (*Escherichia coli* and *Pseudomonas aeruginosa*) and two gram-positive bacteria (*Enterococcus faecalis* and *Bacillus sphaericus*) to UV were assessed. UV caused a complete loss of culturability of these bacteria (Chiang et al., 2022). UV interferes with the transcription and translation of DNA replication by causing pyrimidine bases in DNA to form dimers (Templeton and Traktman, 2022).

In the last three decades, UV-B (280–315 nm) radiation on the Earth’s surface has increased by about 5% over northern mid-latitudes and is predicted to increase until the mid-21st century (Wu et al., 2019). Due to UV radiation, crop yield is projected to decline by 20–25% (Kakani et al., 2003; Barnes et al., 2019). UV radiation, particularly UV-B, has the potential to exert a number of deleterious effects on plants, including the disruption of structure and function of key biomolecules including DNA, proteins, and lipids. Radiation leads to oxidative damage, partial inhibition of photosynthesis, and ultimately a reduction in growth and productivity (Kosobryukhov et al., 2020). UV-B radiation inflicts damage to the photosynthetic apparatus of green plants at multiple sites. For example, the sites of damage include rubisco biosynthesis, oxygen-evolving complex, D1/D2 proteins, light-harvesting complex II, quinones, and cytochrome b (Kataria et al., 2014).

Plants’ leaf surface or phylloplane is the most exposed and vulnerable part to UV radiation. The total area of the global phyllosphere is estimated to be 10^9^ km^2^, twice as large as the surface of the earth, and such space could be colonized by bacterial populations of 10^26^–10^27^ cells, as well as lower numbers of archaea and fungi (Lindow and Brandl, 2003). Therefore, the deleterious effect of UV radiation on the plant will affect all life forms. The phylloplane of plant contains a diverse group of microorganisms, and these organisms are exposed to UV irradiation. Among various microbial groups, the most predominant ones are methylotrophs that are also referred to as pink pigmenting facultative methylotrophs (PPFM). The PPFMs mostly belong to the genus *Methylobacter* are phylogenetically diverse and use one-carbon compounds such as formate, formaldehyde, and methanol, as well as a wide range of multi-carbon substrates as a sole source of carbon and energy (Bennett et al., 2018). They belong to a subgroup of *Proteobacteria*, order Rhizobiales and family Methylobacteriaceae. The unique feature of PPFMs is their ability to oxidize methanol, a methylotrophic property based on the presence of methanol dehydrogenase (*mxaF*) gene (McDonald and Murrell, 1997).

The genus *Methylobacter* comprises 23 species, isolated from a wide range of environments (Hiraishi et al., 1995). While PPFM bacteria on plant surfaces are benefited from methanol produced by plants, these bacteria secrete a variety of auxins and cytokinins that are utilized by the plants leading to an increased plant growth and yield (Klikno and Kutschera, 2017). *Methylobacter* sp. is persistently present in the phyllosphere of numerous plants (Verginer et al., 2010). Methylotrophic bacteria produce 1-aminocyclopropane-1-carboxylate (ACC) deaminase and induce the production of antioxidant enzyme and osmolytes in plants, which helps in mitigating drought/high-temperature stress (Zhang et al., 2021). Methylotrophic bacteria adapt to survive in stress conditions such as low nutrient, drought, and high temperature by producing biofilm and aggregate formation (Krishnamoorthy et al., 2021).

A study suggested that carotenoids produced by microorganisms present in Antarctic render resistance to UV by acting as sunscreen (Agogué et al., 2005). Carotenoids are framed of an important group of isoprenoid chains, characterized by the presence of a conjugated tetraterpene (C_40_) whose colors vary from colorless to yellow, orange, and red. There are over 1,100 such substances found in the terrestrial environment (Yabuzaki, 2017). The conjugated double-bond chain of carotenoids behaves as light-absorbing chromophores playing important biological roles in protecting cells from the damaging effects of UV radiation, as well as exerting antioxidant effects (Wang et al., 2014). However, literature on the role of carotenoid produced from the PPFM on UV protection is limited.

The current study was undertaken with three hypotheses. First, the PPFMs present in the phylloplane of plants are resistant to UV radiation based on the biosynthesis of metabolites including carotenoids. Second, carotenoid biosynthesis occurs in response to UV irradiation. Third, the metabolites can be used to protect other organisms as well as plant from UV radiation. Experiments were conducted with the following objectives: (1) Isolation of a potential PPFM bacterium having potential resistance to UV irradiation; (2) Evaluation of the biosynthesis of carotenoids in response to UV irradiation; and (3) Elucidation of the effect of PPFM and carotenoid on key bacterial species, plant–microbial interaction, and plant growth.

## Materials and methods

### Leaf sample collection

Leaf samples were collected from rice plant (*Oryza sativa*) cultivated at an experimental site located at the Indian Institute of Soil Science (23°30 N latitude, 77°40 E longitude, and 485 m above mean sea level). The location has a semiarid and subtropical climate. It has dry summers and cold winters, with a mean annual air temperature of 25°C, rainfall amounts of 1208 mm, and 56% humidity. It experiences southwestern monsoon rains in July–September. The experimental soil belongs to vertisols, specifically the Hypothermic family of Typic Haplusterts popularly known as “black cotton soil”. The soil contains organic C (5.1 g kg^–1^), available N (215 mg kg^–1^), and available P (2.0 mg kg^–1^), but is high in available K (210 mg kg^–1^). The textural composition of the soil was 15.2% sand, 30.3% silt, and 54.5% clay. Soil’s electrical conductivity (EC) was 0.51 dS m^–1^, and pH was 7.7. Soil pH and EC were measured in a 1:2.5 soil water suspension. Fully grown fresh leaves were collected from 3 randomly selected healthy plants. Individual healthy leaves free from insects and visible dust particles were selected. Samples were stored in plastic bags at 4°C to prevent moisture loss and were brought to the laboratory. Analyses were commenced within 4 h of collection, and sterile procedures were used where appropriate.

### Isolation of ultraviolet-resistant methylotroph bacterium

**FIGURE 1:**
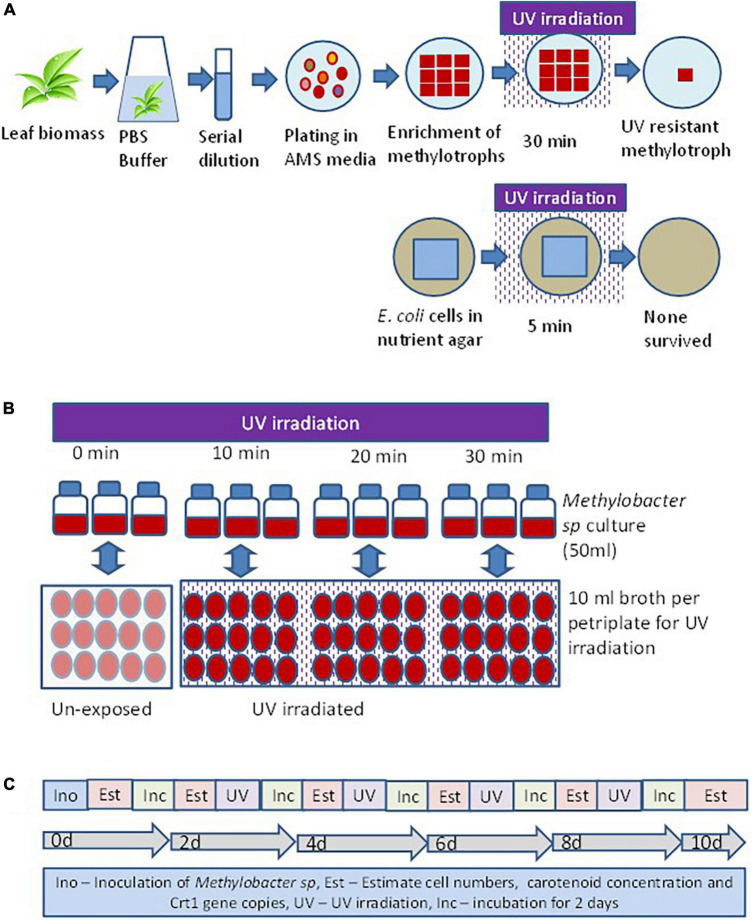
Experimental set up to evaluate the response of methylotroph to UV irradiation. **(A)** Isolation of UV-resistant methylotrophs from the phylloplane of plant leaf biomass. Methylotrophs isolated by enriching leaf surface bacteria in ammonium mineral salt (AMS) agar. Colonies were UV irradiated for 15 min, and the most resistant one was selected for further study. The 16S rRNA gene of the isolate was homologous (>98%) to *Methylobacter sp*. **(B)** Experimental layout for evaluating the effect of UV irradiation on growth of methylotrophs, carotenoid biosynthesis, and Crt1 gene abundance. **(C)** Sequence of steps followed to study the effect of UV irradiation on methylotrophs growth and carotenoid biosynthesis. Ino – Inoculation of isolate; Est – Estimation of cell numbers, carotenoid concentration, and abundance of Crt1 gene copies (only Crt1 was estimated at 0d and 10d); Inc – Incubation of vials; UV – UV irradiation. Broth without UV irradiation served as control, where the petriplates were covered with polypropylene lid to restrict UV radiation. Experiments were conducted in three replicates.

Isolation of methylotrophs was carried out to define the ecological significance of these organisms prevalent on the phylloplane of plants. The method of isolation of a UV-resistant methylotroph is illustrated in Figure 1. Standard protocol was used to isolate methylotrophs following the methodology described elsewhere (Mizuno et al., 2012; Mohanty et al., 2017). Briefly, freshly collected leaf samples were washed with sterile water to remove dust particles settled on the surface of the leaf. The leaf samples (10 g) were suspended in 100 ml of sterile phosphate buffer (120 mmol L^–1^, pH 8) containing (g L^–1^ Na_2_HPO_4_ 1.44; KH_2_PO_4_ 0.24; KCl 0.20; NaCl 8.0; pH 7.4). Flasks were agitated at 150 rpm at 30°C for 2 h in a horizontal shaker. After agitation, 1 ml sample was serially diluted using 9 ml sterile water. Appropriate dilutions were used for inoculation onto ammonium mineral salts agar (pH 7.0) (Whittenbury et al., 1970). The ammonium mineral salts (AMS) medium was sterilized by autoclaving and cooled to 45°C. Filter sterilized vitamin solution (Colby and Zatman, 1973), cycloheximide (to inhibit fungal growth) at 30 μg ml^–1^, and 0.5% (v/v) methanol (carbon source for methylotroph) was added before plating when the temperature of the medium was about 50°C. Plates were incubated at 28 ± 2°C in an incubator (M/s Metrex, N Delhi, India) for 7 days. Bacterial colonies that appeared pink were counted as pink pigmenting facultative methylotroph (PPFM). About 10 such colonies with different colony morphology selected and streaked on to fresh AMS agar plates. Plates were incubated at 28 ± 2°C for 7 days for optimum growth. Nutrient agar (Himedia, Mumbai, India) plates were prepared to grow *E. coli* (JM109, Promega, USA). Plates with *E. coli* were incubated for 2 days for optimal growth. *E. coli* plates were used as control cell. Thirty such plates (6 irradiation time points × 5 replicates) were prepared for both organisms. The time points were exposure duration of 5, 10, 15, 20, 25, and 30 min. Petriplates with fully grown methylotrophs and *E. coli* were UV irradiated in a laminar chamber. The UV lamp was 20 watts, Philips make, wavelength 254 nm, and radiation level 18 mW cm^–2^ sec^–1^. Plates were UV irradiated for different time points (5, 10, 20, and 30 min). After UV irradiation petriplates were covered with aluminum foil for 30 min to minimize shock due to photoreactivation. After UV irradiation, streaks of methylotrophs and *E. coli* were further enriched on respective agar plates. A methylotroph with maximum resistance to UV irradiation was selected for further study.

### Polymerase chain reaction amplification of 16S *rRNA* gene and sequencing

Sequencing of 16S *rRNA* gene of the isolate was performed for taxonomical classification. Colony PCR was performed in a total volume of 50 μl containing cells taken from a freshly grown colony, 1U of Taq DNA polymerase (NEB, USA), 0.2 μM of each primer, and 1 × PCR buffer II (Applied biosystems, CA, USA). The bacterial primers were 8F (5′-AGAG TTT GAT CCT GGC TCA G-3′) and 907R (5′-CCG TCA ATT CMT TTR AGT TT-3′). After the initial denaturizing step of 94°C for 4 min, amplification was carried out with 35 cycles of 94°C for 1 min, 52°C for 30 s, and 72°C for 45 s; final extension carried out at 72°C for 5 min. Amplification was performed on a PCR system (Step one Plus, Applied Biosystem, USA). Amplified PCR product was purified using Geneaid DNA pure kit (Geneaid, Singapore) following the manufacturers’ instruction. The presence and sizes of the PCR amplification product were determined by 1% agarose gel electrophoresis. The purified DNA segment was sequenced separately using 8F and 907R primer using Genetic Analyzer (Applied Biosystems 3130 XL). Both forward and reverse sequences were matched by global alignment, and *contigs* were created by the *CAP contig* assembly tool of *BioEdit ver 7.2.5*. The sequence was inspected manually for the presence of ambiguous base assignments, and chimeric sequences were identified with the Chimera Check program available at NCBI^1^. The sequence was aligned with GenBank database and was matched with the closest relatives. Sequences that were ≥98% similar to one another were considered as a single relatedness group, and the most complete and unambiguous sequences were used for further analysis. Based on the maximum identity score most closely related sequences were selected as most homologous. Sequence was homologous to *Methylobacter sp* and referred to as isolate N39. The 16S *rRNA* gene sequence (∼900 bp) of the isolate (1 number) was deposited in the NCBI database.

### Metabolite extraction, thin-layer chromatography, and high-pressure liquid chromatography analysis

**FIGURE 2:**
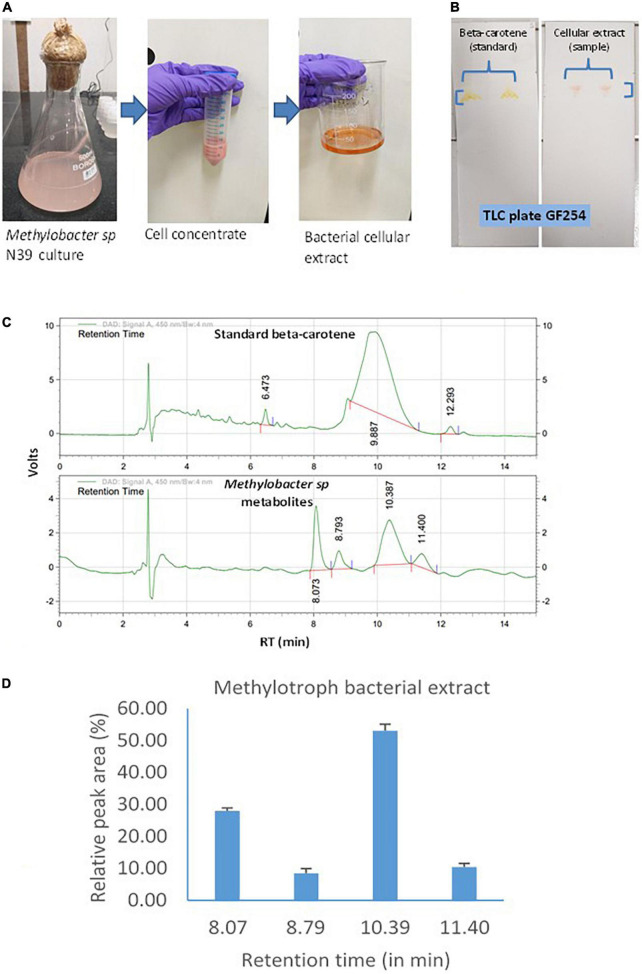
Carotenoid extraction from the methylotroph bacterial isolate (*Methylobacter sp* N39) and qualitative analysis of metabolites or extracts to identify key molecules. **(A)** Extraction of bacterial metabolites. **(B)** Thin layer chromatography (TLC) of the extract to identify the key metabolite. **(C)** High-pressure liquid chromatography (HPLC) analysis of bacterial extract to determine its composition. HPLC chromatograms of standard beta-carotene (top) and metabolites or extracts (bottom). Chromatogram of bacterial metabolite was dominated by a peak representing standard beta-carotene. *X*-axis represents retention time (RT) in minute and Y-axis represents volt. **(D)** Relative area of HPLC peaks of sample (methylotroph bacterial extract). X-axis represents retention time in minutes and Y-axis represents relative peak area (%). Values are arithmetic mean and error bar as standard deviation of three replicated observations.

Cellular extract of the N39 cells analyzed for identifying the major components of the metabolites. Methodology of culturing *Methylobacter sp* N39, extraction of metabolites, and analysis is outlined in Figure 2. Briefly, the methylotroph was grown in 100 serum vials containing 50 ml ammonium mineral salt (AMS) broth as described above. A freshly grown methylotroph colony was suspended in 10 ml sterile water and mixed by vortex. One milliliter of the inoculum was added to 500 ml flasks (3 replicates). Flasks were closed with cotton plug and incubated in a shaker incubator, maintained at 28 ± 2°C and 100 rpm. After 10 days, flasks were taken out from the incubator, and a portion (50 ml) of culture was centrifuged in a falcon tube at 10,000 *g* for 10 min at 4°C. The pellet was suspended in 5 ml methanol and vortex for 10 s. Tubes were centrifuged, and supernatant was transferred to a fresh tube. Extraction procedure was repeated for the second time. Methanolic fraction was evaporated at 70°C to obtain the crude carotenoid and stored at –20°C. For analyzing composition, the extracted pellet was suspended in methanol (80%). Qualitative analysis was carried out by thin-layer chromatography (TLC) and high-pressure liquid chromatography (HPLC). TLC was performed following a standard protocol as described elsewhere (Steiger et al., 2012). The mobile phase was prepared with toluene, ethyl acetate, and methanol at 50:30:20. Based on the literature, we presumed that the pink pigmentation of the methylotroph could be due to carotene (Konovalova et al., 2006; Dourado et al., 2015). Therefore, beta-carotene (Sigma Aldrich, USA) was used as standard for comparison. Retention factor (RF) of standard and sample was estimated as migration distance of substance/migration distance of solvent front (Meyers and Meyers, 2008). Extract was further analyzed by HPLC (Agilant 1220 infinity II LC gradient) fitted with C18 column (4.6 mm × 250 mm, 5 μm, USA) and diode array detector to detect composition of metabolite. Wavelength of detection was 450 nm. Mobile phase was made with acetonitrile and methanol at 70:30 ratio. Flow rate and injection volume were 1.0 ml min^–1^ and 10 μl, respectively. The mobile phase was filtered through a 0.45 μm membrane filter and then denaturized ultrasonically prior to use. All chromatographic operations were conducted at ambient temperature.

### Effect of ultraviolet irradiation on growth of methylotroph, carotenoid biosynthesis, and *CrtI* gene expression

#### Experimental layout and microcosm set-up

Bacterial phytoene desaturase gene (*CrtI*) catalyzes the conversion of the colorless carotenoid phytoene into lycopene. The later acts as the precursor molecule for biosynthesis of beta-carotene carotenoid. Thus, the expression of *CrtI* gene increases the beta-carotene content in cells (Cen et al., 2022). To evaluate *CrtI* gene expression in response to UV exposure, an experiment carried out as illustrated in Figure 1. Twelve serum vials of 100 ml were used in this experiment. Vials were arranged out in a completely randomized design with 4 treatments × 3 replicates. The treatments represented the duration of UV irradiation for 0, 10, 20, and 30 min. Each vial was filled with 50 ml ammonium mineral salt (AMS) broth prepared as described above. A freshly grown methylotroph colony was suspended in 10-ml sterile water and serially diluted. One milliliter of the inoculum was added to each vial. The contents of the vials were mixed well by pipetting, and 1 ml broth was withdrawn to estimate the bacterial abundance and carotenoid concentration. The readings represented values of initial day (0 d). Vials were closed with cotton plug and incubated in a shaker incubator. The vials were maintained at 28 ± 2°C and 100 rpm. After 2 days, vials were taken out from the incubator and a 1-ml sample withdrawn to estimate bacterial abundance and carotenoid concentration. The readings represented values of 2 days. The remaining broth was poured into 5 sterile petriplates (10 cm diameter) at about 10 ml per plate. The depth of the broth on each petriplate was about 1 mm. Plates were irradiated for 0, 10, 20, and 30 min. For the treatment of 0 min, plates were covered with polypropylene lids to stop UV exposure. For other exposure treatments, plates were without a lid during UV exposure. After irradiation, broth from petriplates was poured back into vials and re-incubated. Same procedure of sampling to determine bacterial abundance and carotenoid concentration was followed at 2-day interval representing values of 4, 6, 8, and 10 days. The value of bacterial abundance and carotenoid concentration was plotted to elucidate the role of UV irradiation on methylotroph’s growth and carotenoid biosynthesis.

#### Estimation of bacterial cell abundance and carotenoid concentration

Sample from vials was collected at different incubation period and serially diluted in sterile distilled water. Ammonium mineral salt agar plates were prepared as described above and the abundance of PPFMs was estimated as a number of colony-forming units (CFU) ml^–1^ of broth. Carotenoid was estimated by following the protocol described elsewhere (Reis-Mansur et al., 2019). Briefly, 1 ml broth was taken in a 1.5 ml tube and centrifuged at 10,000 *g* for 10 min at 4°C (Dynamica 14R). The pellet was suspended in methanol and vortex for 10 s. Tubes were centrifuged and the supernatant was transferred to a fresh 2 ml tube. Extraction procedure was repeated for the second time. Methanolic fraction was then evaporated at 70°C to obtain the crude carotenoid and stored at –20°C. For estimating carotenoid, the pellet was suspended in methanol (80%) and measuring absorbance at 450 nm in a UV Vis spectrophotometer. A standard curve prepared using beta-carotene (Sigma Aldrich, USA) of different concentrations and methanol as blank.

#### Real-time polymerase chain reaction o*f CrtI* gene

Broth samples collected at 0 day and 10 days were used to estimate *CrtI* gene copies. DNA was extracted from a 1-ml sample and evaluated in a Biophotometer (Eppendorf, Germany). The PCR reaction mixture (50 μl) contained 20 ng of DNA template, 1U of Taq DNA polymerase (NEB, USA), 0.4 μM of each primer, and 1 × PCR buffer (PCR Buffer II, Applied biosystems, CA, USA). The primers (5’-3’) for *CrtI* gene were CrtIF (AAT ACT TCA AGC CGG TGC TG) and CrtIR (GAC ATG CCG AGG TAC TTG GT) with 186 bp fragment length (Dourado et al., 2013). Thermal cycling profile was set with the initial denaturizing step at 94°C for 4 min, 40 cycles at 94°C for 1 min, annealing temperature for 30 s, and 72°C for 45 s; final extension carried out at 72°C for 5 min. The annealing temperature was 52°C. The PCR amplification products were checked by agarose (1%) gel electrophoresis for the presence and size of PCR band. Fluorescence was measured during the elongation step. Data analysis was carried out with Step one plus software (ABI, USA) as described in user’s manual. The cycle at which the fluorescence of target molecule number exceeded the background fluorescence (threshold cycle [*C*_*T*_]) was determined, from dilution series of target DNA with defined target molecule amounts. The melting curve analysis carried out with temperature increase of 0.3°C per cycle to check the quality of PCR products.

#### Cellular extract of *Methylobacter sp* as ultraviolet protectant

The study was undertaken to evaluate the effect of cellular extract of *Methylobacter sp* N39 on extending UV protection to *E. coli*. Experiment was laid out in completely randomized design with 4 treatments, 7 UV irradiation timings, and 3 replicates. The 4 treatments were only *E. coli* cells (control), *E. coli* cells + commercial beta-carotene at 10 μg ml^–1^, *E. coli* cells + cellular extract of N39 containing beta-carotene at 10 μg ml^–1^, and *E. coli* cells + N39 cells. A total of 84 LB agar plates were prepared for this experiment. *E. coli* dh5α cell was used as a reference cell, which was multiplied in LB broth at 37°C in a rotary shaker incubator for 2 days. Cell numbers were enumerated in terms of OD 600. One milliliter culture was added to a 25-ml falcon tube containing 5 ml normal saline (0.8% NaCl). Four such tubes were prepared for the treatments. The content of each tube was mixed well, and 0.1 ml was spread inoculated on LB agar plate. Plates were UV irradiated for 0 (without irradiation), 5, 10, 15, 20, and 30 min. After exposure, plates were incubated at 37°C for 48 h and number of colonies counted. Survival fraction was estimated as N/N_0_, where N_0_ was the number of colonies developed from 0 min irradiated plates, and N was the number of colonies that appeared from irradiated plates. Rate of inhibition was estimated as the slope of survival fraction over irradiation time.

#### Scanning electron microscopy

Cellular structure was examined with and without carotenoid extract. The experiment had 4 treatments comprising (1) N39 cells without radiation, (2) N39 cells with 15 min UV radiation, (3) *E. coli* cells with 5 min UV radiation, and (4) *E. coli* cells mixed with carotenoid extract radiated for 5 min. *E. coli* cells were mixed with carotenoid extract at 10 μg ml^–1^. A fixative solution was added to the bacterial cells, containing 0.25% glutaraldehyde and 10 mM phosphate buffer saline (PBS) buffer (pH 7.4) for overnight incubation. The fixed sample was rinsed thrice with PBS buffer and followed by dehydration with ascending concentrations of 10, 20, 30, 60, 80, and 100% ethanol for 10 min each. After dehydration, the samples were dried at room temperature for 2 h. Sterilized coverslips were placed and allowed bacterial cells to attach to their surfaces. A scanning electronic microscope (Carl Zeiss SMT Ltd., Zeiss EVO 18) was used to obtain the electron micrographs of cells. The microscope was equipped with a BSD detector and tungsten electron source. The electron high tension (EHT) was in the range of 200 V to 30 kV, and the image resolution was 50 nm.

## Effect of *Methylobacter sp* and carotenoid extract on ultraviolet resistance of rhizobium and effectiveness toward legume growth

Experiment was carried out in a completely randomized design with 2 seed types, 5 treatments, and 3 replicates. Seeds of pigeon pea (*Cajanus cajan* var Asha) and soybean (*Glycine max*) were surface sterilized following the standard method (Mohanty et al., 2017). *Rhizobium* (*Bradyrhizobium japonicum*) cells were multiplied in yeast extract mannitol broth (Jorrin et al., 2021), and *Methylobacter sp* N39 was multiplied in ammonium nutrient broth as described above. Cells were harvested and diluted in sterile distilled water to make suspensions of 10^8^ cells ml^–1^. Carotenoid extract of N39 cells was prepared with a concentration of 10 μg ml^–1^ as described above. Seeds (75 g) of each plant type were divided into 5 equal parts, each representing one treatment. The five treatments were (1) control (no cells and no UV), (2) + *Bradyrhizobium japonicum* 10^6^ cells g^–1^ (no UV), (3) + *Bradyrhizobium japonicum* 10^6^ cells g^–1^ (UV irradiation), (4) + *Bradyrhizobium japonicum* of 10^6^ cells g^–1^ + N39 cells (10^6^ cells g^–1^) (UV irradiation), and (5) + *Bradyrhizobium japonicum* of 10^6^ cells g^–1^ + carotenoids (10 μg g^–1^) (UV irradiation). Seeds with cells or carotenoids were mixed well and air-dried for 1 h. Seeds of each treatment were divided into 3 equal parts representing three replicates. Seeds were placed on petriplates, evenly distributed and UV irradiated for 15 min as per treatment. Those without UV irradiation were covered with polypropylene lids to prevent irradiation. Subsequently, seeds were sown in plastic pots filled with soil. Plants were grown in a plant growth chamber with 12 h light, 28 ± 2°C, and 60% moisture (Genesys, India). On the 30th day after sowing, plants were harvested to estimate number of nodules and acetylene reduction assay.

### Acetylene reduction assay

Nodules (1 g) from plants were placed into 50 ml serum vials. Headspace of the vials was injected with 10% acetylene. Vials were incubated for 4 h at 28 ± 2°C. Headspace ethylene concentration was measured in a gas chromatograph (GC) (CIC, Baroda, India) equipped with flame ionization detector (FID) and porapak Q column (1/8 in id, 3 m length). The GC was set at oven temperature 60°C, injector 110°C, and detector 150°C. Under the set condition, a retention time of ethylene was 5.5 min.

### Effect of *Methylobacter sp* on ultraviolet resistance of plant

The experiment was conducted to examine how the *Methylobacter sp* N39 and its carotenoid extract protect plant from UV irradiation. Experiment laid out in a completely randomized design with 3 foliar treatments, 3 UV irradiation doses and 5 replicates. The foliar treatments were spraying of sterile water, cells of N39, and carotenoid extract of N39. The UV irradiation was for 0.5 h d^–1^, 1 h d^–1^, and 1.5 h d^–1^ for 3 consecutive days (Sztatelman et al., 2015). Briefly, seeds of pigeon pea (*Cajanus cajan* var Asha) were surface sterilized with 1% sodium hypochlorite for 5 min and washed three times with distilled water (Mohanty et al., 2017). Four seeds were sown in plastic pots containing sterilized sand with five replicates. Hoagland’s solution was added to meet the nutrient requirement (Van Delden et al., 2020). After 30 days of sowing, foliar spraying was carried out with 10 ml of bacterial suspension (10^6^ CFU ml^–1^), carotenoid extract (10 μg ml^–1^), or sterile distilled water. After spraying, plants were allowed to air dry and then UV (254 nm, 20 w) irradiated for 30 min, 1 h, and 1.5 h. Irradiation was done for three consecutive days. On 40th day of sowing, plants were harvested to determine chlorophyll A content (Vernon, 1960) and dry weight following standard protocols.

### Statistical analysis

Arithmetic mean and standard deviation for different parameters were estimated to define the variation of parameters in response to various experiments. Linear models are estimated to exhibit the relationship between different parameters. All statistical analysis was carried out using Microsoft Excel 2010.

## Results

### Isolation of ultraviolet-resistant methylotroph

The abundance of pink pigmenting facultative methylotrophs (PPFM) varied from 5–25 × 10^5^ CFU g^–1^ leaf biomass. Out of 10 PPFM, one survived 30 min of UV irradiation. *E. coli* cells were completely killed within 5 min of UV irradiation. The 16S rRNA gene sequence of the methylotroph was homologous (99%) to *Methylobacter sp*, referred to as isolate N39. The 16S rRNA gene sequence of the isolate was submitted to GenBank (www.ncbi.nlm.nih.gov) with accession number MW276130.

### Metabolite extraction, TLC, and HPLC analysis

Methylotroph bacterial metabolites were extracted and analyzed to identify the key biomolecules. Extraction and analysis of extracts under normal growth conditions were assayed by TLC and HPLC (Figure 2). TLC was performed to know whether beta-carotene was the main component of the bacterial extract. This was determined by estimating the retention factor (RF) of both sample and standard (beta-carotene). RF was the ratio of distance traveled by a compound and solvent within a specified time. RF value of both the standard and sample was 0.98, which indicated that the methylotroph extract was mainly constituted of beta-carotene. Subsequently, a sample was analyzed by HPLC to determine the composition of the methylotroph metabolite. The key question was whether the bacterial extract contained only beta-carotene or was a mixture of different compounds. Retention time of standard beta-carotene was 9–10 min. In the case of sample (extract), there were 4 peaks with retention times 8.073, 8.793, 10.3, and 11.4 min. Beta-carotene was detected at 10.3 min. Relative area of peaks was estimated to determine the most dominant peaks in the extract. Peak of beta-carotene (RT 10.3 min) contributed 53%, while the peak at 8.073 min contributed 27.9% of total peak area. Peaks at 8.793 contributed 8.5% and that of 11.4 min contributed 10.45%. In this analysis, sample was extracted from a methylotroph culture containing 0.06 ± 0.005 μg carotenoid ml^–1^ broth having 10^6^ cells.

### Growth rate, carotenoid biosynthesis, and *CrtI* gene abundance

**FIGURE 3:**
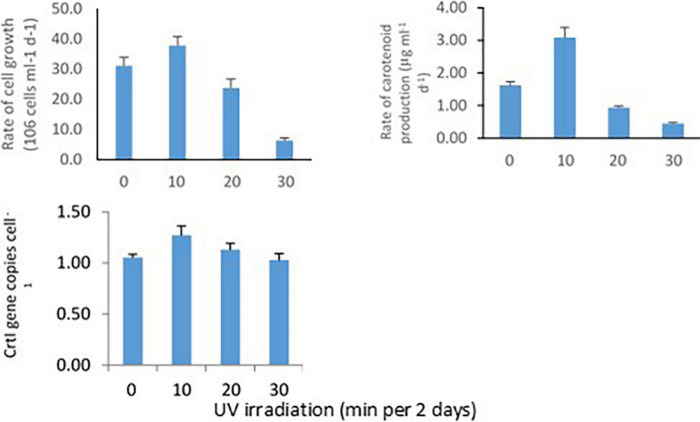
Effect of UV irradiation on cellular activities of *Methylobacter sp* N39. The cellular activities were rates of carotenoid biosynthesis, growth rate, and carotenoid biosynthesis gene (CrtI) copy number. *Methylobacter sp* N39 was grown in ammonium mineral salt broth. Broth was exposed to UV irradiation for 0, 10, 20, and 30 min every 2 days. After exposure broth was re-incubated allowing cells to grow and biosynthesize carotenoid. The process of exposure was repeated at 2 days interval for 10 days. Cell numbers **(top left)** and carotenoid **(top right)** concentration were estimated at 2 days interval. Rates were estimated as slope of values over incubation period. After completion of incubation period (10 days), DNA was extracted from broth to quantify carotenoid biosynthesis gene, CrtI by real-time PCR using SYBR green chemistry **(bottom left)**. *X*-axis represents UV irradiation (min in 2 days). *Y*-axis represents different parameters. Each data point represents arithmetic mean and standard deviation as error bar of three replicated observations.

*Methylobacter sp* N39 was grown in ammonium nutrient salt broth and UV irradiated for 0, 10, 20, and 30 min every 2 days. This setup was to determine the rate of bacterial cell growth, carotenoid biosynthesis, and *CrtI* gene abundance in response to UV irradiation (Figure 3). Rate of cell growth rate (10^6^ cells ml^–1^ broth d^–1^) was 31.2, 37.9, 23.8, and 6.4 in 0, 10, 20, and 30 min of irradiation. Rate of carotenoid production (μg ml^–1^ d^–1^) varied from the lowest of 0.45–3.09. The lowest was in 30 min irradiation and highest in 10 min irradiation. The abundance of *CrtI* gene copies (numbers cell^–1^) varied from 1.03 to 1.27. *CrtI* gene copies in N39 cells were highest in 10 min irradiation and lowest in 30 min irradiation.

### Response of ultraviolet irradiation on growth and carotenoid secretion of *Methylobacter sp*

**FIGURE 4:**
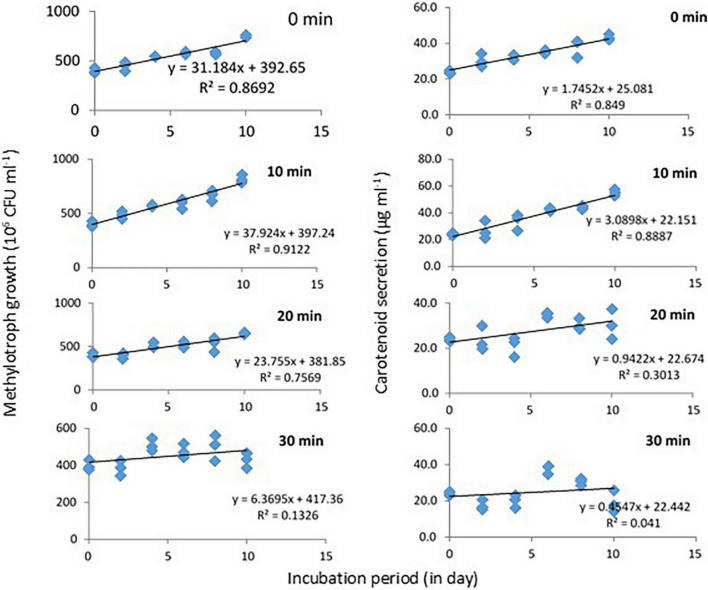
Growth and carotenoid production by a pink pigmenting *Methylobacter sp* N39 (Genbank Acc no MW276130.1) in response to UV radiation. The bacterium was cultured in a synthetic medium. Broth was exposed to UV radiation for different timings (0, 10, 20, and 30 min). For each data point, a portion of broth was sampled at 2 days interval and used for estimating cell abundance and carotenoid concentration. Rest of the broth was exposed to UV radiation and incubated till next sampling. *X*-axis represents the incubation period, while *Y*-axis represents cell abundance **(left panel)** and carotenoid concentration **(right panel)**. Values are presented in 4 replicates.

Response of *Methylobacter sp* N39 to UV irradiation was evaluated in terms of growth and carotenoid production (Figure 4). Initial bacterial density was 4 × 10^8^ CFU ml^–1^ in all treatments. Cultures were UV irradiated for 0, 10, 20, and 30 min at 2 days intervals. The values of bacterial abundance and carotenoid concentration were plotted against the incubation period. After 10 days, bacterial count was maximum (816 × 10^6^ CFU ml^–1^) in the treatment of 10 min UV irradiation. Growth rate in this treatment followed a linear model of *y* = 37.924x + 397.24, *r*^2^ = 0.912, where x represented incubation period. Growth rate decreased with further UV irradiation and was lowest in 30 min irradiation. Carotenoid concentration initially was 24 μg ml^–1^ and increased to a maximum of 55 μg ml^–1^ in the 10 min irradiated treatments. Carotenoid secretion declined with higher UV irradiation. Carotenoid biosynthesis was linearly modeled as y = 3.0898x + 22.151, r^2^ = 0.8887, where x was incubation period.

### Effect of extract of *Methylobacter sp* on ultraviolet resistance of *Escherichia coli*

**FIGURE 5:**
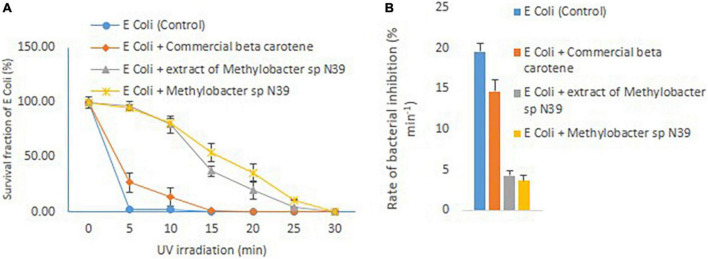
Effect of *Methylobacter sp* N39 on extending UV resistance to *E. coli* bacteria. Cells of *E. coli* were multiplied in LB media and prepared for different treatments and UV irradiation. The treatments were (1) *E. coli* suspension (control), (2) *E. coli* + commercial beta-carotene at 10 μg ml^–1^, (3) *E. coli* + extracts of *Methylobacter sp* N39 extract containing 10 μg beta-carotene ml^–1^, and (4) *E. coli* + *Methylobacter sp* N39. Cell suspensions were spread plated on LB plates and UV irradiated for 0, 5, 10, 15, 20, 25, and 30 min. Colonies were counted after incubation. Left panel **(A)** represents survival fraction (%) of *E. coli* in response to UV irradiation. *X*-axis represents irradiation time in minutes, and *Y*-axis represents the survival fraction. Right panel **(B)** represents the inhibition rate of *E. coli* in response to UV irradiation. Rate of inhibition is determined from the slope of survival fraction vs irradiation time. Each value represents arithmetic mean ± standard deviation as error bar of three replicated observations.

The effect of cellular extract of *Methylobacter sp* N39 on enhancing the UV resistance to *E. coli* was evaluated. The result of survival fraction and the rate of inhibition by different treatments are presented in Figure 5. The survival of *E. coli* against UV irradiation was increased in the trend of *E. coli* + cells of *Methylobacter sp* N39 > *E. coli* + beta-carotene of *Methylobacter sp* N39 > *E. coli* + commercial beta-carotene > *E. coli* cells alone. None of *E. coli* cells survived 5 min of UV irradiation. Survival fraction reduced to 0% in 5 min. About 20% of cells were killed min^–1^ by UV irradiation. Amendment of commercial beta-carotene extended tolerance to UV. Survival fraction was extended till 10 min. The rate of decline of the population of *E. coli* was 14.67% min^–1^. The survival of *E. coli* was significantly enhanced by the treatment of either beta-carotene or cells of *Methylobacter sp* N39. *E. coli* cells tolerated irradiation for 30 min in both cases. The rate of decline of *E. coli* cells was 4.30% min^–1^ in the case of cellular beta-carotene extract and 3.67% min^–1^ in the case of additional *Methylobacter sp* N39 cells.

### Scanning electron microscopy

**FIGURE 6:**
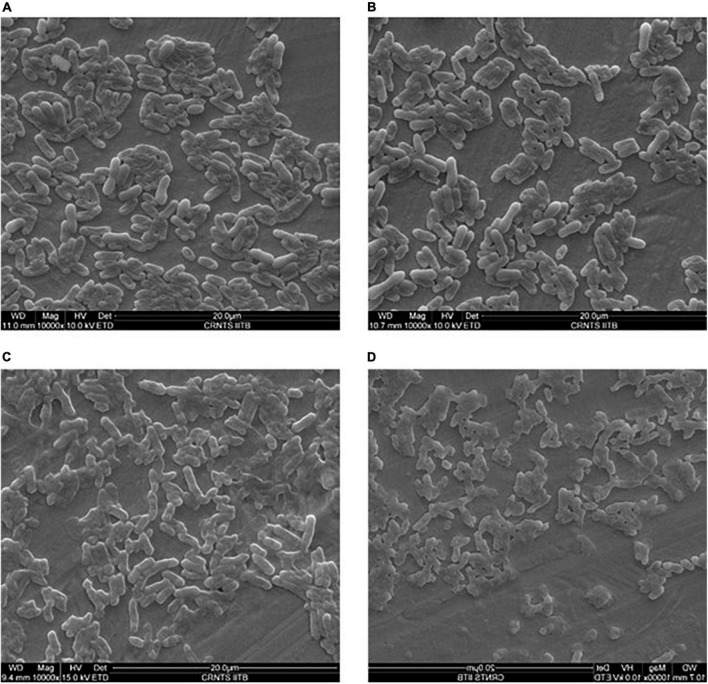
Scanning electron micrograph of bacterial cells under UV radiation and the effect of carotenoid extract on protecting cells from radiation. *Methylobacter sp* N39 cells without UV exposure **(A)**; *Methylobacter sp* N39 cells after 15 min of UV exposure. Cells survived UV radiation without any apparent disruption of cellular structure **(B)**. *E. coli* cells exposed to UV C radiation for 5 min **(C)**. The cellular structure damaged exhibiting deformed cells due to UV radiation; **(D)** *E. coli* cells treated with crude carotenoid after exposure to UV C radiations for 5 min. *E. coli* cells were protected from UV radiation by the carotenoid extract of *Methylobacter sp* N39 cells.

UV radiation on cellular structure was evaluated by electron microscopy. Cell wall of *E. coli* cells was disrupted after 5 min of exposure to UV radiation (Figure 6). Whereas cell wall of *E. coli* cells treated with crude carotenoid remained intact even after UV light exposure. It infers that carotenoid pigment successfully protected the cells from UV radiation. Control cells of *Methylobacter sp* N39 without UV treatment shows intact cell wall. However, cell structure remained intact even after 15 min exposure to UV radiation. It could be inferred from these images that isolate N39 shows strong resistance against UV radiations.

### Effect of *Methylobacter sp* and carotenoids on protecting *rhizobium* toward plant growth promoting attributes

**FIGURE 7:**
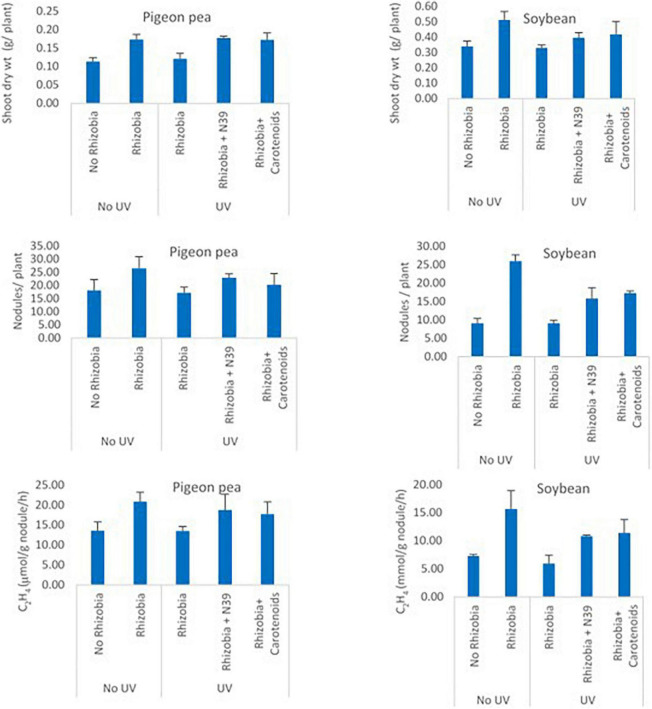
Effect of *Methylobacter sp* N39 and carotenoid extract on plant–rhizobium interaction in response to UV irradiation. Seeds of pigeon pea and soybean were prepared with the following treatments. 1 - control (no rhizobium no UV), 2 - rhizobium no UV, 3 - rhizobium with UV, 4 - rhizobium + *Methylobacter sp* N39 with UV, and 5 - rhizobium + carotenoid extract with UV. After treatments seeds were air dried and UV irradiated for 15 min. Seeds were planted and grown under controlled condition. Plants were harvested after 30 days of sowing and parameters (shoot dry weight, number of nodules, and acetylene reduction assay) were estimated. *Y*-axis represents parameters, and *X*-axis represents treatments. Values are presented as arithmetic mean ± standard deviation of three replicated observations.

The effect of *Methylobacter sp* N39 and carotenoids on *rhizobium* was evaluated from the plant parameters including shoot dry weight, number of nodules per plant, and acetylene reduction assay (Figure 7). Shoot dry weight (g plant^–1^) varied from 0.11 to 0.17 in pigeon pea and 0.33–0.51 in soybean. Number of nodules varied from 17.25 to 26.56 in pigeon pea and 9.16–26 in soybean. Acetylene reduction or ethylene (C_2_H_4_) production (μmol g^–1^ nodule h^–1^) varied from 13.51 to 20.89 in pigeon pea and 5.98–15.67 in soybean. The plant parameters varied similarly in response to treatments irrespective of plant type. The values of plant parameters were highest in the treatment of *rhizobium* alone and lowest in no *rhizobiu*m control. The positive effect of *rhizobium* was nullified with UV irradiation as the *rhizobium* cells were most likely killed by UV. However, the addition of *Methylobacter sp* N39 or carotenoid extract nullified the negative effect of UV irradiation. The effect of *Methylobacter sp* N39 and carotenoids had a similar effect on plant parameters.

### Effect of *Methylobacter sp* and extract on ultraviolet resistance of plant

**FIGURE 8:**
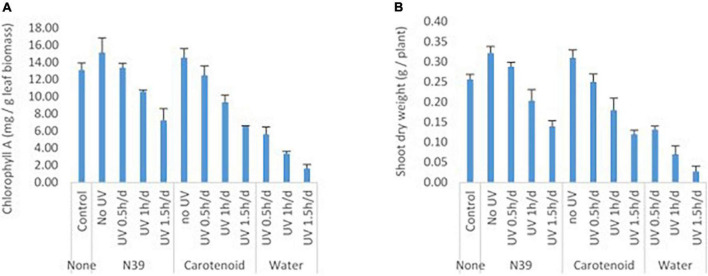
Effect of foliar application of cells of *Methylobacter sp* N39 and carotenoid on plant’s UV resistance. The test plant Pigeon pea (*Cajanus cajan* var Asha) was grown under controlled condition. On 30th day of sowing, plants were subjected to foliar application of *Methylobacter sp* N39 (10^6^ cells/ml), carotenoids extracted (10 μg/ml) from *Methylobacter sp* N39, and water as control. The treated plants were UV irradiated for 0.5, 1, and 1.5 h per day for 3 consecutive days. On 40th day of sowing, plants were analyzed for **(A)** chlorophyll A content and **(B)** shoot dry weight. *X*-axis represents treatments and *Y*-axis represents parameters. Values are presented as arithmetic mean with error bar as standard deviation of three replicated observations.

Foliar application of cells of *Methylobacter sp* N39 and carotenoid extracts enhanced plants’ resistance to UV irradiation (Figure 8). Plant parameters including dry weight and chlorophyll content were increased by the foliar application of either product. Chlorophyll content varied from 1.61 to 15.17 mg g^–1^ leaf, while the dry weight of shoot biomass varied from 0.03 to 0.32 g per plant. UV irradiation significantly affected the plant parameters. There was about a 48–89% decline in plant dry weight and a 57–88% decline in chlorophyll content in plants without bacterial or carotenoid spray. However, the negative effect of UV decreased by 45% in dry weight and 42% in chlorophyll content by spraying cells of N39. The negative effect of UV was declined by 36% in dry weight and 38% in chlorophyll content by spraying carotenoid extract. However, the extent of UV protection was high at lesser dose (0.5 h) of UV irradiation. For example, the negative effect of UV on dry weight can be reduced to 60% by N39 spraying and 50% by carotenoid spraying.

## Discussion

The study aims to evaluate the mechanism of UV resistance exhibited by the phylloplane-dwelling pink pigmenting facultative methylotroph (PPFM) and demonstrate the effect of metabolites on rendering UV resistance to itself and other organisms. Pink pigmenting facultative methylotrophs are found in soil and other plant parts of plants and are characterized by plant growth-promoting attributes, but the role of these PPFMs on UV resistance is less explored. An abundance of PPFM in the phylloplane estimated were in the range of 5–25 × 10^3^ CFU g^–1^ fresh leaf. Similar values have been observed in other plants including white clover (*Trifolium repens*) (Corpe and Rheem, 1989), vegetable plants (Mizuno et al., 2012), pea (Macey et al., 2020), and other plants like grass (Aydogan et al., 2020). Methylotrophs were isolated using ammonia mineral salt media with methanol as a carbon source as methanol is preferred carbon source of methylotrophs. PPFMs were UV irradiated for 5, 10, 20, and 30 min to isolate the UV-resistant ones. Similar approach has been used to study the effect of UV irradiation on the phyllosphere microbial community of lettuce (Jacobs and Sundin, 2001). *E. coli* cells were killed within 5 min of UV radiation. A similar observation has been reported where *E. coli* cells were killed within 1–2 min of UV (254 nm) irradiation (Lindell et al., 1996; Wright et al., 2000).

The methylotroph that survived 15 min of UV radiation was identified as M*ethylobacter sp* (Krishnamoorthy et al., 2018). This bacterium has been found in other plants. For example, 30 different methylotrophic bacterial isolates were found in 15 groundnut genotypes. Among all, *M. sp* enhanced root growth and improved the formation of root hair to a maximum level. *M. sp* has been isolated from the seeds of the medicinal plant *Combretum erythrophyllum.* Various gamma radiation–resistant pink pigmenting Methylobacterium have been isolated from soils of the Republic of Korea. The isolates were homologous to *M. currus, M. aquaticum, M. platani, M. frigidaeris, M. terrae*, and *M. organophilum* (Kim et al., 2020). A methylotroph bacterial strain *Methylobacterium sp* 17Sr1-43 isolated from soil was resistant to both gamma and UV-C irradiation. Complete genome sequence annotation showed the presence of the genes involved in radiation resistance (Kang and Srinivasan, 2018). However, literature on the response of *Methylobacter* to UV irradiation is limited. The current study highlights the resistance of *Methylobacter* to UV-A radiation.

It is reported that many pink pigmenting *Methylobacterium* biosynthesize beta-carotene that represents xanthophylls, particularly hydroxy carotenoids (Konovalova et al., 2006). However, studies are limited to denote the composition of pigment extract. To unravel the composition, the extracted pigment was analyzed by TLC and HPLC. TLC indicated that the pink pigment was mainly composed of beta-carotene, as the retention factor (RF) of both extract and beta-carotene was the same. However, to exhibit the other components of extract, samples were analyzed by HPLC. There were 4 different peaks. Beta-carotene was the main component, constituting 53% of total peak area. The 2nd most dominant peak observed at 8.07 min was 27% of total peak area. Other two peaks constituted about 10% of total peak area. There was about 1.63 μg carotenoid production per 10^6^ cells per day resulted to 16.3 μg of carotenoid in 10 days. A similar value has been observed in a study on carotenoid production from different *Methylobacterium*. Quantitative content of carotenoids was 0.45 mg g^–1^ of dry weight for *M. fujisawaense* and *M. mesophilicum* and 0.23 mg g^–1^ for *M. extorquens*. The cellular pigment extract of *Methylobacterium* strains contained about 80% of carotenoids (Konovalova et al., 2007).

The experiment was carried out to examine how bacterial cell growth, carotenoid biosynthesis, and *CrtI* gene abundance occurred in response to UV irradiation. This was undertaken mainly to unravel whether UV irradiation enhances carotenoid biosynthesis. UV irradiation of 10 min stimulated cellular growth, carotenoid biosynthesis, and *CrtI* gene expression but inhibited with a longer period of irradiation. Linear models of bacterial growth and carotenoid biosynthesis indicated that both parameters linearly increased in the treatment of 10 min irradiation. The r^2^ values were high in 10 min irradiation treatment and lowest in 30 min irradiation. Based on the result, it is presumed that the *Methylobacter sp* N39 produces carotenoid more when a small dose of UV irradiation is given. It is possible that carotenoid biosynthesis gene was stimulated by UV irradiation, in this case, 10 min exposure enhanced carotenoid biosynthesis. Higher carotenoid concentration resulted into higher cellular growth. However, longer UV irradiation of 20 min and 30 min inhibited carotenoid biosynthesis thereby declined cellular growth.

The study was aimed to elucidate how the methylotroph bacterium *Methylobacter sp* N39 extends UV resistance to other bacteria. *E. coli* was taken as a reference organism as it is one of the common bacteria and well-studied organism. Experiment was taken up with two key questions. First, whether the beta-carotene extract of *Methylobacter sp* N39 was better than the commercial ones. Second, whether the cells of *Methylobacter sp* N39 also extend UV resistance. To answer the key questions, experiment was laid out with 4 different treatments comprising only *E. coli* cells, *E. coli* cells + commercial beta-carotene, *E. coli* cells + beta-carotene from *Methylobacter sp N39*, and *E. coli* cells + *Methylobacter sp N39*. *E. coli* cells under the treatments were spread plated on LB agar plates and UV irradiated for 30 min. In the first treatment, where *E. coli* cells were plated alone, cells were killed within 5 min of irradiation. Similar results have been reported earlier (Vermeulen et al., 2008; Holck et al., 2018). Commercial beta-carotene extended UV resistance to 10 min and declined mortality rate by 15%. However, beta-carotene produced by *Methylobacter sp* N39 decreased the mortality rate to less than 5%. There are few indirect studies that support this finding. In a study on biofilm communities, it was observed that organisms present on the outer layer protect the organisms present in an inner layer from UV radiation. The first few top layers of microbial cells produce specialized compounds such as mycosporine-like amino acids and carotenoid pigments (de Carvalho, 2017). A similar result was observed with *E. coli*, when the cells were grown with 0.1?mg ml^–1^ or 1?mg ml^–1^ pigment extract of *Microbacterium* sp. *E. coli* cells were protected from UV irradiation which indicated that the resistance of *Microbacterium* sp. against UV-B radiation was connected with photoprotection by its pigments (Reis-Mansur et al., 2019). The treatment of *E. coli* + *Methylobacter sp* N39 also declined the mortality of *E. coli* cells to 3.87%. It is likely that the intracellular pigment and other biomolecules absorbed UV radiation most efficiently than simple bacterial extract. HPLC analysis exhibited multiple compounds present in the bacterial extract. A similar result has been observed in the case of *Microbacterium* sp. The cellular extract of the bacterium contains alpha-carotene, echinenone, canthaxanthin, and astaxanthin, which have multiple beneficial effects for UV protection (Reis-Mansur et al., 2019). Carotenoids protect cells in multiple ways including protection against oxidative stress, adaptation to temperature, and fluidity of cell membrane (Seel et al., 2020).

Scanning electron microscopy indicated that *Methylobacter sp* N39 and its carotenoid secretion can protect *E. coli* cells from UV radiation. *E. coli* cells were killed by 5 min of UV radiation due to disruption of cell membrane. Whereas the cellular structure of N39 cells remained intact even after 15 min of UV radiation. Crude extract of N39 cells could protect *E. coli* cells even after 5 min of UV radiation. Probably, the carotenoid extract absorbed the radiation and protected cells.

The experiment on rhizobial cell was undertaken to decipher how *Methylobacter sp* N39 and carotenoids protect *Bradyrhizobium japonicum* from deleterious effect of UV irradiation. Two legumes (pigeon pea and soybean) were evaluated to examine the consistency of the result. Seeds were coated with a *B. japonicum*, and the parameters were crop biomass, number of nodules, and acetylene reduction assay (ARA), which were indicative of plant-rhizobial interaction. For example, higher rhizobial abundance was represented as a higher nodule number, N_2_ fixation (ARA activity) leading to higher crop biomass. Seeds were coated with *B. japonicum* alone, or along with cells of *Methylobacter sp* N39 or carotenoids. Coated seeds were exposed to UV irradiation for 15 min. The positive effect of rhizobial cell inoculation has been reported to increase soybean biomass (Aserse et al., 2020), nodule numbers (Ntambo et al., 2017; Li et al., 2018), and ARA activity (Nguyen et al., 2020). Similar results have been observed in pigeon pea (Araujo et al., 2020). Result highlighted that plant parameters increased three folds in terms of fresh weight, nodule number, and ARA activity in the treatment of seeds coated with *B. japonicum*. Plant parameters in the treatment of seeds + *B. japonicum* + UV irradiation were at par with control (no *B. japonicum* coating). Data suggested that UV irradiation killed all coated *B. japonicum* cells, resulting the values at par with no coated control. However, the parameters in the treatment of + *B. japonicum* + *Methylobacter sp* N39 or + carotenoids were at par with the treatment of + *B. japonicum*. Result suggested that *Methylobacter sp* N39 as well as carotenoid equally contributed in protecting *rhizobial* cells from UV irradiation. It is likely that coated rhizobial cells were protected. Carotenoids contain pigments like beta-carotene which absorbed UV irradiation and nullified the negative effect of UV. Similarly, the cells of *Methylobacter sp* N39 contain different pigments in addition to beta-carotene which absorbed UV irradiation and protected *B. japonicum* from UV more efficiently.

Experiment was carried out to determine the extent of the beneficial effect of *Methylobacter sp* N39 and carotenoid extract on protecting plant from UV irradiation. Bacterial cells and carotenoid extracts were applied as foliar application as previously evaluated (Saa et al., 2015). Application of *Methylobacter sp* N39 and carotenoid extract enhanced plant biomass and chlorophyll content. *Methylobacterium* has been used as a microbial inoculant to improve crop productivity due to its plant growth–promoting attributes (Grossi et al., 2020). However, there are less studies on the beneficial effect of carotenoids. UV irradiation of 1.5 h per day for 3 days had a maximum negative effect on the plant parameters. Plant biomass was severely affected due to UV irradiation as most of the leaves were shredded. Dry weight of plant biomass was declined by 89% and chlorophyll by 87% over the un-irradiated control. The inhibition effect was reduced to 35–45% by N39 and 35–49% by carotenoid spraying. Carotenoid extracts mainly contain beta-carotene which absorbs UV irradiation. In the case of bacterial cell, it contains pigments that absorbed UV irradiation and extended protection against UV. Carotenoids are constituted of isoprenoid chains, characterized by the presence of a conjugated tetraterpene (C_40_) (Yabuzaki, 2017). The conjugated double-bond chain of carotenoids behaves as light-absorbing chromophores playing important biological roles in protecting cells from the damaging effects of UV radiation, as well as exerting antioxidant effects (Wang et al., 2014). Foliar application of cells was better in terms of reducing the negative effect of UV irradiation than the carotenoid extracts itself. It could be by three possibilities: first, the cell contains complex biomolecules including a range of pigments that have not been separated by the simple alcoholic extraction. The complex biomolecules have a higher potential in absorbing UV irradiation than the extract. Second, carotenoid attains activated state during UV irradiation and returns to original state easily enabling it to absorb higher doses of irradiation. Third, carotenoid quenches reactive oxygen species produced during stress, preventing damage from UV irradiation (Niedzwiedzki et al., 2017).

## Conclusive remarks

The current project was undertaken to explore pink pigmenting methylotroph to alleviate an abiotic stress caused by UV radiation for agricultural use. In this context, a potential pink pigmenting methylotroph bacterium *Methylobacter sp* N39 was isolated. Carotenoid extract of the bacterium was dominated by beta-carotene which was the key biomolecule for UV resistance. The bacterium produced beta-carotene at a rate (μg ml^–1^ d^–1^) of 0.45–3.09. Biosynthesis of beta-carotene was stimulated by exposing the bacterium N39 to UV radiation for 5 min per day. The significance of N39 cells and carotenoid extract on UV protection was evaluated by examining the effect on *E. coli, rhizobium* (*Bradyrhizobium japonicum*), and plant. Studies on the effect of carotenoid on UV resistance of *E. coli* concluded that the life span of *E. coli* can be extended from 5 to 30 min by adding N39 cells or carotenoids. Carotenoid extract of N39 cells protected *E. coli* cells from cell disruption under UV radiation. Similarly, the N39 cells and carotenoid extract protected legume (pigeon pea and soybean)- *rhizobium (B. japonicum)* interaction under UV irradiation. Spraying of N39 cells and carotenoids on leaf also protected pigeon pea from UV irradiation. This study highlights that the isolate *Methylobacter sp* N39 as well as the carotenoid extract of the bacterium can be explored to alleviate UV radiation stress for improving agriculture.

## Data availability statement

The datasets presented in this study can be found in online repositories. The names of the repository/repositories and accession number(s) can be found below: https://www.ncbi.nlm.nih.gov/genbank/, MW276130.

## Author contributions

SM conceptualized the experiment and wrote the manuscript. HM and AB carried out the experiment. GD carried out molecular studies. RP, NA, and MD assisted in plant sample analysis. BK analyzed the data and contributed in interpretation. DJ, AS, and AP contributed in discussion. All authors contributed to the article and approved the submitted version.

## Footnotes

1
https://www.ncbi.nlm.nih.gov/
